# Myelodysplasia-associated mutations in serine/arginine-rich splicing factor SRSF2 lead to alternative splicing of CDC25C

**DOI:** 10.1186/s12867-016-0071-y

**Published:** 2016-08-23

**Authors:** Lindsey Skrdlant, Jeremy M. Stark, Ren-Jang Lin

**Affiliations:** 1 Department of Molecular and Cellular Biology, Irell & Manella Graduate School of Biological Sciences, Beckman Research Institute of the City of Hope, Duarte, CA 91010 USA; 2 Department of Cancer Genetics and Epigenetics, Irell & Manella Graduate School of Biological Sciences, Beckman Research Institute of the City of Hope, Duarte, CA 91010 USA

**Keywords:** SRSF2, Myelodysplastic syndromes, RNA splicing, CDC25C, DNA damage response

## Abstract

**Background:**

Serine–arginine rich splicing factor 2 (SRSF2) is a protein known for its role in RNA splicing and genome stability. It has been recently discovered that SRSF2, along with other splicing regulators, is frequently mutated in patients with myelodysplastic syndrome (MDS). The most common MDS mutations in SRSF2 occur at proline 95; the mutant proteins are shown to have different RNA binding preferences, which may contribute to splicing changes detected in mutant cells. However, the influence of these SRSF2 MDS-associated mutations on specific splicing events remains poorly understood.

**Results:**

A tetracycline-inducible TF-1 erythroleukemia cell line was transduced with retroviruses to create cell lines expressing HA-tagged wildtype SRSF2, SRSF2 with proline 95 point mutations found in MDS, or SRSF2 with a deletion of one of the four major domains of the protein. Effects of these mutants on apoptosis and specific alternative splicing events were evaluated. Cells were also treated with DNA damaging drugs for comparison. MDS-related P95 point mutants of SRSF2 were expressed and phosphorylated at similar levels as wildtype SRSF2. However, cells expressing mutant SRSF2 exhibited higher levels of apoptosis than cells expressing wildtype SRSF2. Regarding alternative splicing events, in nearly all examined cases, SRSF2 P95 mutants acted in a similar fashion as the wildtype SRSF2. However, cells expressing SRSF2 P95 mutants had a percent increase in the C5 spliced isoform of cell division cycle 25C (CDC25C). The same alternative splicing of CDC25C was detected by treating cells with DNA damaging drugs, such as cisplatin, camptothecin, and trichostatin A at appropriate dosage. However, unlike DNA damaging drugs, SRSF2 P95 mutants did not activate the Ataxia telangiectasia mutated (ATM) pathway.

**Conclusion:**

SRSF2 P95 mutants lead to alternative splicing of CDC25C in a manner that is not dependent on the DNA damage response.

**Electronic supplementary material:**

The online version of this article (doi:10.1186/s12867-016-0071-y) contains supplementary material, which is available to authorized users.

## Background

**Fig. 1:**
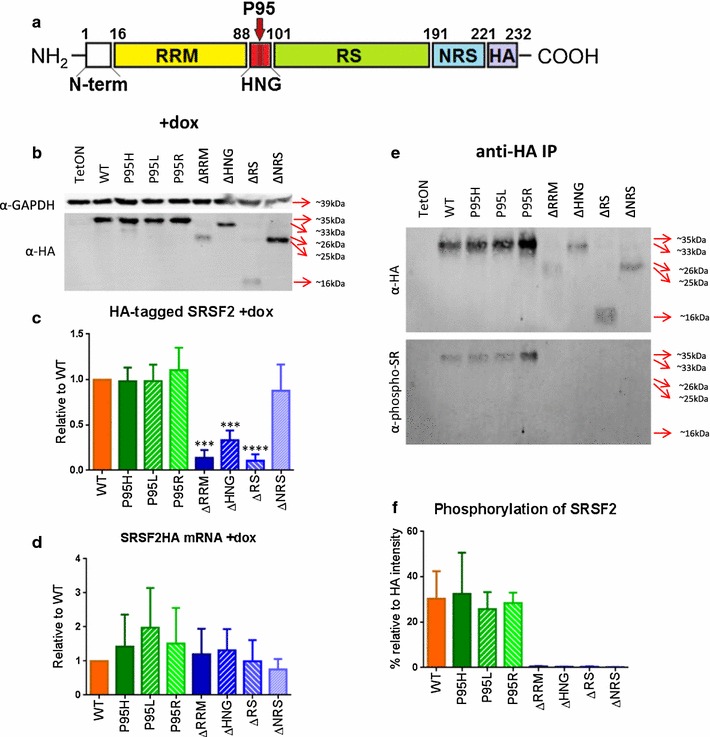
Expression and phosphorylation of SRSF2 P95 and deletion mutants. **a** Schematic of SRSF2HA ORF showing the primary protein domains removed to create SRSF2 deletion mutants. *RRM* RNA recognition motif; *HNG* hinge region; *RS* arginine/serine-rich domain; *NRS* nuclear retention signal; *HA* hemagglutinin tag. Numbers denote amino acids defining the domain boundaries within the full length protein. Location of the P95 amino acid within the hinge region is denoted with a *large red arrow*. **b** Western blot of protein isolated from cell lines after 48 h doxycycline induction. **c** Average HA-tagged protein expression in induced cell lines, normalized to GAPDH expression. Statistics are based on one-way analysis of variance (ANOVA) with comparison to SRSF2^WT^ cell line (n = 5). **d** qRT-PCR analysis of HA-tagged SRSF2 mRNA expression, normalized to SRSF2^WT^. No statistical significance using one-way ANOVA with comparison to SRSF2^WT^ (n = 4). **e** Western blot using anti-HA and anti- phospho-SR antibodies after immunoprecipitation of HA-tagged SRSF2 protein. **f** Analysis of phospho-SR signal normalized to HA-tagged protein signal. Statistics use one-way ANOVA with comparison to SRSF2^WT^ (n = 3). *p* values: *≤0.05; **≤0.01; ***≤0.001; ****≤0.0001

Serine–arginine rich splicing factor 2 (SRSF2), previously named SC35, is a member of the SR protein family of splicing regulators. The primary role of SR proteins is to regulate splice site selection for both constitutive and alternative splicing. In addition to their role in splicing, SR proteins are also involved in the maintenance of genome stability through the prevention of R-loop structure formation during transcription [1–4]. Structurally, SRSF2 can be divided into specific protein domains (Fig. 1a). The RNA recognition motif (RRM) is involved in binding to exonic or intronic splicing enhancers in the nascent pre-mRNA transcript. The eponymous serine–arginine rich domain (RS) is primarily involved in protein–protein interactions with other spliceosomal components and with the 7SK complex involved in transcriptional elongation [3, 5, 6]. In addition, serine phosphorylation at the RS domain significantly affects the subnuclear localization and functions of SRSF2 [7–11]. The short amino acid sequence between the RRM and RS domains is the hinge region (HNG). The exact function of this region is largely unknown, though studies with SRSF1 (ASF/SF2) suggest that it may have an important role in kinase docking [12]. The last major domain of SRSF2 is the nuclear retention signal (NRS) at the C-terminal end of the protein, which is unique to SRSF2. Deletion of this NRS allows SRSF2 to shuttle between the nucleus and cytoplasm, similar to the shuttling function of other SR proteins that aid in mRNA export [9].

It was recently discovered that SRSF2 is mutated in 10−15 % of patients with Myelodysplastic syndrome (MDS) and 25–30 % of patients with chronic myelomonocytic leukemia (CMML) [13–19]. Both of these diseases are aging-associated hematopoietic disorders that occur primarily in individuals over the age of 60 [20]. The only effective long term treatment for either disease is a bone marrow transplant, which is often not possible to perform due to both the age of the patients and a high relapse rate in patients with advanced disease [21–24]. While the cause of these disorders is still unknown, alternative splicing in genes related to hematopoiesis and cell cycle regulation, such as CDC25C and RUNX1, have been found in patients with MDS or AML [25, 26]. For both of these diseases, patients with SRSF2 mutation have a miscoding of the proline at position 95 (P95) to a histidine, arginine, or leucine during the early stages of the disease. These mutations persist throughout the disease [18, 27]. Recent research has also shown that P95 mutations of SRSF2 affect the ability of SRSF2 to bind its canonical splicing enhancer sequences in RNA [28, 29]. In addition, the P95H mutation of SRSF2 can increase death of hematopoietic cells and cause changes in hematopoiesis [28, 30]. Results from these studies support the notion that these mutations of SRSF2 are involved in MDS pathogenesis. However, the mechanism for how these mutations lead to disease development is still unknown.

We have constructed stable cell lines expressing from a Tet-inducible promoter HA-tagged wildtype SRSF2 (SRSF2^WT^), HA-tagged SRSF2 with point mutations found in patients with MDS (SRSF2^P95H^, SRSF2^P95L^, and SRSF2^P95R^), and HA-tagged SRSF2 with in-frame deletions of each of the four major domains of the protein (SRSF2^ΔRRM^ = deletion of the RNA recognition motif, SRSF2^ΔHNG^ = deletion of the hinge region, SRSF2^ΔRS^ = deletion of the arginine/serine-rich domain, and SRSF2^ΔNRS^ = deletion of the nuclear retention signal) in TF-1 erythroleukemia cells (Fig. 1a). Our data showed that while the SRSF2^P95R/L/H^ mutations did not affect cellular localization of the protein, they did increase early apoptosis and affect the alternative splicing of CDC25C towards a shorter isoform (CDC25C-C5) that has previously been shown to be upregulated when DNA is damaged in breast cancer cells exposing to sub-lethal levels of doxorubicin and cisplatin [31]. Interestingly, we found SRSF2 mutant induced alternative splicing of CDC25C does not require activation of the DNA damage response pathway that is activated with cisplatin treatment.

## Methods

### Plasmid construction

The pRevTRE-SC35HA (SRSF2HA) tet-inducible plasmid was a gift from Xiang-Dong Fu’s lab at UCSD [2]. Mutations of P95 were produced using site-directed mutagenesis with the QuikChange Kit (Agilent) with the following primers: SRSF2-P95X sense (5′-CTACGGCCGCCDCCCGGACTCAC-3′) and SRSF2-P95X antisense (5′-GTGAGTCCGGGHGGCGGCCGTAG-3′), where D is A, T, and G and H is A, T, and C [32]. SRSF2 deletion mutants were produced using the InFusion cloning system (Clontech) and primers that overlapped the deletion sites: ΔRRM F1 (ATGTGGAGGGTATGACCTCCATGGCGCGCTACGGC), ΔRRM R1 (GCCGTAGCGCGCCATGGAGGTCATACCCTCCACAT), ΔHNG F1 (CGAGCTGCGGGTGCAAAGCCGCCGGGGACC), ΔHNG R1 (GGTCCCCGGCGGCTTTGCACCCGCAGCTCG), ΔRS F1 (CCCGGACTCACACCACCCTCCCCCAGTGTCCA), ΔRS R1 (TGGACACTGGGGGAGGGTGGTGTGAGTCCGGG), ΔNRS F1 (GGTCTCGGTCCAGGAGTCTCGAGTACCCATACGACG), ΔNRS R1 (CGTCGTATGGGTACTCGAGACTCCTGGACCGAGACC). SRSF2HA gene sequence was confirmed using Sanger sequencing. The lentiviral plasmid for expression of the reverse Tet transactivator protein (pHIV7-rtTA-V15), the plasmids for lentiviral packaging (pC-GP, pCMV-rev, pCMV-G), and the plasmids for retrovirus packaging (pCMV-GP and pCMV-G) were gifts from the lab of Jiing-Kwan Yee at City of Hope.

### Production and transduction of lentivirus and retrovirus

For the rtTA lentivirus, 293T cells were transfected with pHIV7-rtTA, pC-GP-2, pCMV-rev2, and pCMV-G using calcium phosphate [32]. After 48 h, cell supernatant was collected, filtered through a 0.45 µm filter, and precipitated in a 10 % PEG solution overnight at 4 °C. Virus was then concentrated by centrifuging the PEG solution 30 min at 2000×*g*. Supernatant was decanted and virus pellet was resuspended in the remaining solution. Virus was tittered on HT1080 cells by G418 resistance. TF-1 cells were transduced with rtTA lentivirus at an MOI of 0.003 for 48 h and then diluted to single cells and plated in 96-well plates for G418 selection. Single clones were collected and assayed for doxycyline induction potential. Clone #6 was chosen as the TF-1 TetON parental cell line.

For the SRSF2HA retroviruses, 293T cells were transfected with the respective pRevTRE-SRSF2HA plasmid, pCMV-GP, and pCMV-G sing calcium phosphate transfection. After 48 h, virus was collected as above. Virus was tittered on HT1080 cells by hygromycin B selection. TF-1 TetON cells were transduced with resulting retroviruses at an MOI of 0.1 for 48 h. Each group was then selected for 2 weeks using hygromycin B to produce the TF-1 TetON SRSF2HA cell lines.

### cDNA synthesis and RT-PCR

1 × 10^6^ cells were pelleted and resuspended in 100 µl Trizol. After 5 min incubation, 100 μl 100 % EtOH was added and RNA was isolated using Direct-zol kit (Zymo Research). 1 µg RNA was used for cDNA synthesis using either SuperScript III RT (Invitrogen) or PrimeScript RT (Clontech). Each RT-PCR reaction contained 10 μl 2× Taq (BioPioneer), 0.5 µM each primer, and 1 μl cDNA. Primer list and reaction conditions can be found in Additional file 1: Table S1.

### Western Blot Analysis

1 × 10^6^ cells were washed 1× with PBS and then resuspended in 50 µl dH20. 50 µl 2× Laemmli buffer, pre-warmed to 85 °C, was added to each sample. Samples were incubated 10 min at 95 °C. Samples were then aliquoted and flash frozen in liquid nitrogen to be stored at −80 °C. For SDS-PAGE, samples were diluted 3.5-fold using 1× Laemmli buffer, and 7 μl was loaded per lane. Following SDS-PAGE, proteins were transferred to PVDF membrane. The membranes were blocked for 1 h at RT with either 5 % (w/v) nonfat dry milk in TBST (50 mM Tris–Cl, pH 7.5. 150 mM NaCl, 0.1 % Triton X-100) or 3 % (w/v) BSA in TBST. Membranes were washed 3× with TBST. Primary antibodies [rabbit anti-HA tag (Abcam; ab9110; 1:10,000), mouse anti-GAPDH (Invitrogen; ZG003; 1:5000), mouse anti-SR proteins [1H4] (Santa Cruz Biotech; sc-13,509; 1:200), rabbit anti-CDC25C [E303] (GeneTex; GTX61135; 1:500), rabbit anti-phospho CHK2 (Cell Signaling Technology; 2661; 1:2000), and mouse anti-phospho p53 [16G8] (Cell Signaling Technology; 9286; 1:500)] were diluted as stated in TBST and incubated overnight at 4 °C. Membranes were washed 3× with TBST. Secondary antibodies [goat anti-rabbit IgG-IRDye 680LT (Li-Cor; 926-68021; 1:20,000), goat anti-mouse IgG-IRDye 800CW (Li-Cor; 926-32,210; 1:20,000), goat anti-rabbit-HRP (BioRad; 170–6515; 1:3000), and goat anti-mouse-HRP (BioRad; 170–6516; 1:3000)] were diluted as stated in TBST and incubated with the membranes for 4 h at RT. Membranes were washed 3×, 5 min each wash, and subsequently scanned with an Odyssey machine (for Li-Cor secondaries) or exposed to X-ray film (for HRP secondaries).

### Immunoprecipitation

1 × 10^7^ cells were pelleted for 5 min at 400×*g*. Supernatant was removed and cells were resuspended in a buffer containing 50 mM HEPES (pH 7.8), 3 mM MgCl_2_, 300 mM NaCl, 1 mM DTT, 0.1 mM PMSF, 1X Halt™ Protease Inhibitor Cocktail (Thermoscientific), 5 mM NaF, 2 mM NaVO_3_, 0.5U/µl DNase I, and 5U/µl benzonase. Samples were incubated at 4 °C for 1 h with constant rotation, after which samples were centrifuged for 3 min at 4 °C, 10,000×*g*. Supernatant was added to 50 μl anti-HA agarose beads (Sigma) and total volume was brought to 200 μl with TBS. Samples were incubated overnight at 4 °C with constant end-over-end rotation. Agarose beads were pelleted for 10 s at 16,000*g*, and supernatant was removed as the unbound fraction. Pellet was washed 5× with TBST. Samples were eluted by incubating agarose beads with 50 µl 1 mg/ml HA peptide (ThermoFisher Scientific) at 30 °C for 15 min. Eluate was assayed by Western blot.

### Immunofluoresecence

Glass coverslips (Fisher Scientific; 22 × 22–1.5) were incubated with 0.01 % poly-l-lysine (Sigma) for 10 min. Poly-l-lysine solution was removed and coverslips were air-dried. 2 × 10^5^ cells were incubated on coverslips 1 h at 37 °C. Coverslips were washed 2× with PBS. Cells were fixed by incubating coverslips with 4 % (w/v) paraformaldehyde in PBS for 20 min at RT. Coverslips were then washed 2× with PBS and incubated 15 min at RT with 0.1 % (v/v) Triton X-100 in PBS. Cells were blocked by incubating coverslips overnight at 4 °C in 10 % (v/v) FBS in PBS. Coverslips were incubated with primary antibodies [rabbit anti-HA tag (Abcam; ab9110), mouse anti-SC35 (Abcam; ab11826), and mouse anti-γH2AX (ab11174)] each diluted 1:100 in the blocking solution for 1 h at RT. Coverslips were washed 5× with PBS and then incubated 1 h at RT with secondary antibodies [goat anti-rabbit IgG-AF647 (Jackson ImmunoResearch; 111-605-003) and goat anti-mouse IgG-AF488 (Jackson ImmunoResearch; 115-545-003)] diluted 1:100 in the blocking solution. Coverslips were then washed 5× with PBS and incubated 10 min at RT with 1× CellMask Orange (LifeTechnologies). Coverslips were then washed 3× with PBS and mounted on glass slides using VectaShield Hardset Mounting media with DAPI (Vector Laboratories). Slides were incubated overnight at 4 °C before imaged using a Zeiss LSM 700 confocal microscope. Analysis for HA-tagged protein localization was done using Image-Pro Plus (Media Cybernetics). The whole cell area was outlined in the Cell Mask Orange channel image and copied into the HA protein channel image. The nucleus was outlined in the DAPI channel image and also copied into the HA protein channel image. The integrated optical density (IOD) was analyzed independently for the whole cell and nucleus. The IOD for the cytoplasm alone was calculated by subtracting the IOD for the nucleus from the IOD of the whole cell.

### Apoptosis assay

1 × 10^5^ cells were washed 1× with PBS. Cells were stained for 15 min using FITC-Annexin V and PI from FITC Annexin V Apoptosis Kit I (BD Pharmigen). Samples were then analyzed on a Cyan FACS machine. Cells were considered to be apoptotic based upon positive FITC detection. The stage of apoptosis was determined by the level of PI staining of the FITC-positive cells. Early apoptosis lacked any PI staining. Middle apoptosis had positive, albeit low levels of, PI staining. Late apoptosis was defined by high levels of PI staining.

### Cell proliferation assay

5 × 10^6^ cells were stained on day 0 in 10 µM CFSE (carboxyfluorescein succinimidyl ester, Thermo Fisher Scientific) for 15 min at 37 °C. Each day including day 0, half of the cell culture was removed from the flask, washed 2× with PBS, fixed for 15 min in 4 % (w/v) paraformaldehyde in water, washed 2× with PBS, and stored at 4 °C in the dark. After all time points for a single biological replicate were collected, samples were analyzed on a Cyan FACS machine. Data are plotted by using CFSE log mean.

### Cell cycle analysis

1 × 10^6^ cells were washed 2× with PBS. Cells were resuspended in 500 µl ice-cold 80 % EtOH and incubated overnight at 4 °C. Cells were washed 2× with PBS, and resuspended in 0.25 % Triton X-100, 20 µg/ml PI (propidium iodide), and 0.1 ng/ml RNase A. Cells were incubated overnight at 4 °C. Samples were analyzed on a Cyan FACS machine. Data were analyzed using the cell cycle tool in FlowJo v6.

## Results

### Characterization of SRSF2-HA-expressing cell lines

We started our study by engineering a TF-1 TetON cell line. The TF-1 cell line was chosen due to its classification as an erythroblast, allowing for inducible expression of an HA-tagged SRSF2 in a cell type similar to those commonly affected in MDS patients in order to examine phenotypic changes that may occur as a result of SRSF2 mutations. Furthermore, by PCR and Sanger sequencing, none of the common MDS-related mutations in *U2AF1(S34)*, *SF3B1(K700)*, or *SRSF2(P95)* were detected in the parental TF-1 cell line (data not shown).

We used site directed mutagenesis to produce eight different SRSF2 constructs: wildtype (SRSF2^WT^), each of the three MDS- and CMML-related point mutations (SRSF2^P95H^, SRSF2^P95L^, and SRSF2^P95R^), and four deletions for each of the primary domains of SRSF2 for comparison (SRSF2^ΔRRM^, SRSF2^ΔHNG^, SRSF2^ΔRS^, and SRSF2^ΔNRS^) (Fig. 1a).

In order to assay for transgene expression, each of the SRSF2 cell lines (WT, P95H, P95L, P95R, ΔRRM, ΔHNG, ΔRS, and ΔNRS) and the parental TF-1 TetON cell line were cultured for 48 h, both with and without induction using 2 μg/ml doxycycline. After 48 h, protein and RNA were isolated from each line. Western blotting using anti-HA antibodies detected the expression of HA-tagged proteins with the expected size for each construct upon doxycycline induction (Fig. 1b). A small amount of the HA-tagged protein was expressed from the minimal CMV promoter without doxycycline induction (Additional file 2: Figure S1). The expression levels for SRSF2^ΔRRM^, SRSF2^ΔHNG^, and SRSF2^ΔRS^ were noticeably lower than others (Fig. 1b, c). qRT-PCR of the ectopically expressed SRSF2-HA mRNAs showed no significant differences in the RNA level between the WT and mutants (Fig. 1d). Therefore, it appears that a deletion of the RRM, HNG, or RS domain negatively affects protein stability and/or efficient translation.

Since the hinge region of SRSF1 is required for kinase docking [12], we wanted to determine whether the disease-related P95 mutations of SRSF2, which occur in the hinge region, would affect the phosphorylation level of SRSF2. To this end, we immunoprecipitated the HA-tagged SRSF2 using an antibody against the HA tag. Western blots of the immunoprecipitated samples showed that SRSF2^P95H^, SRSF2^P95L^, and SRSF2^P95R^ were phosphorylated to the same level as SRSF2^WT^, as measured by anti-phospho-SR antibody (1H4 monoclonal antibody) while none of the deletion mutants showed detectable levels of phosphorylation (Fig. 1e, f).

**Fig. 2:**
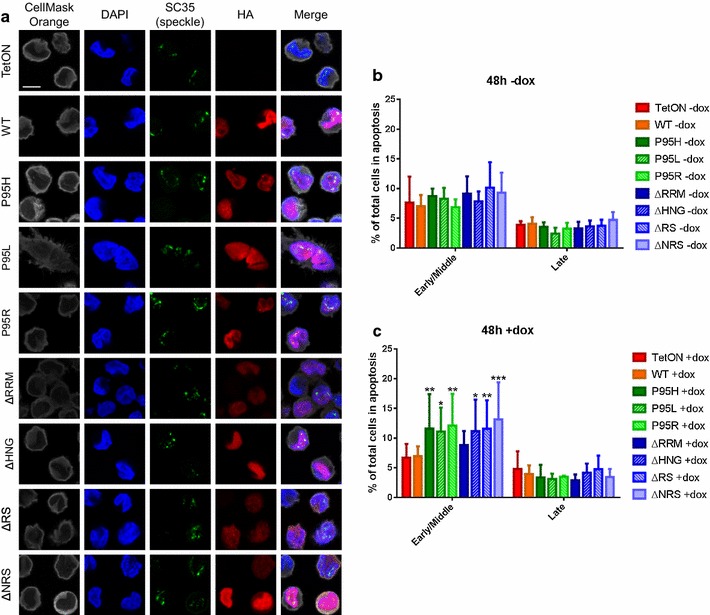
Apoptosis and subcellular localization of P95 and deletion SRSF2 mutants. **a** Representative immunofluorescence images of SRSF2HA cell lines (n = 3). CellMask Orange stained cytoplasm, DAPI stained DNA in the nucleus, anti-SC35 speckle marker stained SC35/SRSF2 in the nuclear speckles, anti-HA stained HA-tagged SRSF2 protein. **b** Average percentage of cells in early or late apoptosis at 48 h in the absence of doxycycline induction. **c** Average percentage of cells in early or late apoptosis at 48 h post-doxycycline induction. Statistics use two-way ANOVA with comparison to TF-1 TetON (n = 4). *Asterisks* are *p* values as in Fig. 1

As stated previously, unlike other SR proteins that are shuttled between the nucleus and the cytoplasm, SRSF2 is confined to the nucleus [9]. To examine whether any of the mutations would affect the cellular localization of SRSF2, we stained the tet-induced cells with anti-HA antibodies. Immunofluorescence results from the anti-HA staining showed that SRSF2^WT^, SRSF2^P95H^, SRSF2^P95L^, SRSF2^P95R^, SRSF2^ΔRRM^, and SRSF2^ΔHNG^ were all confined to the nucleus (Fig. 2a; Additional file 3: Figure S2a). SRSF2^ΔNRS^ was located in both the nucleus and cytoplasm in agreement with previous studies showing that deletion of the NRS allows SRSF2 to shuttle between the nucleus and cytoplasm [9]. Unexpectedly, SRSF2^ΔRS^ was also present in both the nucleus and cytoplasm when it was expected to be located only in the cytoplasm due to loss of the primary nuclear localization signal [9, 33–35]. It is possible that the remaining presence of the NRS allows for partial nuclear localization. We also stained each of the cell lines with an anti-SC35 antibody that specifically stains the nuclear phosphorylated SC35 speckles [33, 36]. Previous research has shown that the phosphorylation state determines whether SRSF2/SC35 is localized within nuclear speckles [10]; therefore the pan-nuclear staining of HA-tagged SRSF2 indicated that a substantial portion of the HA-tagged protein is not in a phosphorylation state reserved for speckle localization. We also observed nuclear SC35-speckles with similar sizes and numbers among the cell lines, indicating the expression of the SRSF2-HA proteins did not significantly alter the formation or the dynamics of nuclear speckles (Fig. 2a).

Factors that can initiate an apoptotic signaling cascade, such as Fas, Fas-L, tumor necrosis factor-a, and tumor necrosis factor-related apoptosis initiating ligand, are upregulated in bone marrow cells of MDS patients [37, 38], and it is thought that this increase in apoptosis in the bone marrow may contribute to the cytopenias that are a hallmark of MDS [39]. To assay our cell lines for changes in apoptosis, FITC-Annexin V and propidium iodide (PI) staining were used to determine apoptosis levels in each of the TF-1 cell lines (Additional file 3: Figure S2b). Without doxycycline induction, there was no significant difference in apoptosis levels between any of the cell lines (Fig. 2b). However, with the doxycycline-induced expression of any of the three P95 mutant SRSF2 transgenes, cells showed a significant increase in early/middle apoptosis as compared to the SRSF^WT^ and parental TetON cell lines (Fig. 2c). This result suggests that SRSF2 P95 mutations may be pro-apoptotic. We also noticed that SRSF2^ΔHNG^, SRSF2^ΔRS^, and SRSF2^ΔNRS^ expressing cells also showed an increase in early/middle apoptosis as compared to the control cell lines, whereas SRSF2^ΔRRM^ showed no effect on apoptosis. We have also measured cell proliferation using CFSE staining. Upon staining, CFSE covalently binds intracellular molecules such as proteins, and as the cells divide the existing CFSE-bound proteins are redistributed to the daughter cells resulted in decreasing CFSE staining per cell. Thus, cell proliferation can be accurately measured by monitoring CFSE staining in cell culture [40]. By using CFSE staining, we did not observe significant change of cell proliferation with any of the SRSF2 mutants (Additional file 3: Figure S2c). Thus, increased apoptosis in SRSF2^P95H/L/R^ is consistent with increased apoptosis of MDS cells.

### Alternative splicing changes

**Fig. 3:**
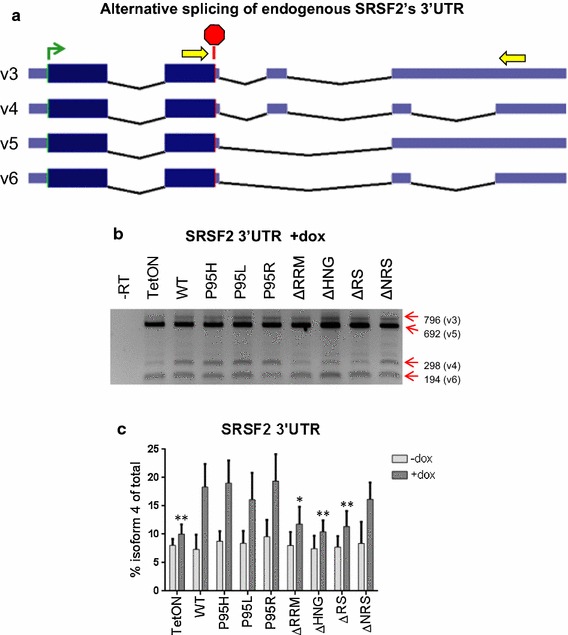
Alternative splicing at the 3′UTR of endogenous SRSF2 transcripts. **a** Schematic of previously described SRSF2 isoforms (splice variants 3, 4, 5, and 6) that result from alternative splicing of the 3′UTR. *Yellow arrows* depict general location of primers used in RT-PCR. **b** Representative gel from RT-PCR of endogenous SRSF2′s 3′UTR after 48 h treatment with 2 μg/ml doxycycline. The sizes in base pairs of the PCR products corresponding to the splice variants are labeled on the right. **c** *Bar graphs* depicting changes in  % isoform 4 (v4) of total RNA. Statistics are based on two-way ANOVA with comparison to SRSF2^WT^ (n = 4). *Asterisks* are *p*values as in Fig. 1; note that the data were compared to the WT and not to the tetON

SRSF2 is known to regulate alternative splicing of a number of pre-mRNAs including its own [41]. There are six splice variants of SRSF2 described in the database, and four of them were detected by RT-PCR in TF-1 cells using the primers depicted (Fig. 3a, b). Among these, splice isoforms v3, v4, and v6 are substrates of nonsense-mediated decay (NMD) because they all have at least one intron that is more than 50 nucleotides downstream from the stop codon (Fig. 3a) [42]. The splice variant 5 (v5) has an intron 7 nucleotides downstream from the stop codon, which does not activate NMD, and is the predominant RNA species (Fig. 3b). As previously shown [41], SRSF2^WT^ overexpression promoted inclusion of alternative exons and resulted in an increase in isoform 4 expression as detected by RT-PCR (Fig. 3b, c). Similarly, expression of a P95 point mutant (SRSF2^P95H^, SRSF2^P95L^, or SRSF2^P95R^) also led to an increase of v4, indicating that these P95 mutations of SRSF2 do not affect its ability to auto-regulate its exon inclusion. On the contrary, expression of the RRM, RS, or HNG deletion mutants did not cause a significant increase of v4, while expression of SRSF2^ΔNRS^ did (Fig. 3c). This supports previous findings that deletion of the NRS does not affect the alternative splicing function of SRSF2, whereas both the RRM and RS domains are required for this function [9, 33, 43–45].

We next investigated whether any of the SRSF2 mutants would alter splicing of other mRNAs that have been previously shown to be affected by SRSF2. To begin with, E2F1-mediated overexpression of SRSF2 has been shown to promote alternative splicing of a set of apoptosis-related genes to their pro-apoptotic form [46]. We assayed alternative splicing of BCL-X, caspase-8, and caspase-9 by RT-PCR. At 48 h post-induction, we did not observe significant changes in alternative splicing in any of the SRSF2-expressing TF-1 cell lines (Additional file 4: Figure S3). Next, we assayed splice variants of BAP1 and TRA2A, which are found to change in mouse fibroblasts upon SRSF2 knock out [47]. We observed no significant splicing changes in these genes with doxycycline-induced overexpression of SRSF2^WT^ or any of the SRF2 mutants in TF-1 cells (Additional file 5: Figure S4).

**Fig. 4:**
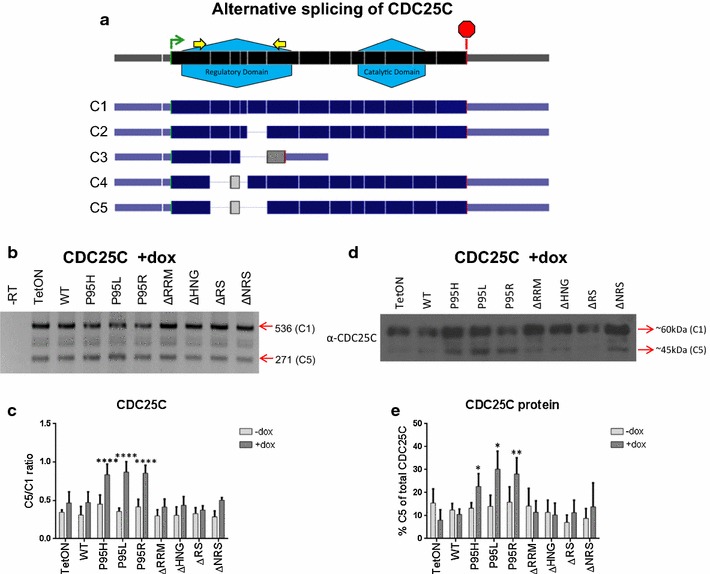
Alternative splicing of CDC25C in SRSF2 mutant cell lines. **a** Schematic of previously described five CDC25C isoforms.* Black* is the CDC25C RNA with all 14 exons (the exon boundaries are marked). The general locations for the N-terminal regulatory domain and the C-terminal catalytic domain, as well as the RT-PCR primers, are annotated. *Dark blue* is the known CDC25C alternative splicing transcripts. *Gray* depicts areas of frame shift that occurs with alternative splicing change in C3, C4, and C5. **b** Representative gel from RT-PCR of CDC25C after 48 h treatment with 2 μg/ml doxycycline. The sizes in base pairs of the PCR products corresponding to the splice variants are marked on the right. **c** *Bar graphs* depicting ratio of C5/C1 isoforms. Statistics are based on two-way ANOVA with comparison to SRSF2^WT^ (n = 5). **d** Western blot depicting CDC25C protein expression in the presence of doxycyline induction. **e** Graph depicting the Western results shown in D for CDC25C protein expression at 48 h post-induction. Statistics are based on two-way ANOVA with comparison to SRSF2WT (n = 5). *Asterisks* are *p* values as in Fig. 1

We then analyzed the alternative splicing of CDC25C, a dual-specific phosphatase critical for the G2/M checkpoint pathway of the cell cycle. CDC25C has previously been found to be alternatively spliced in the bone marrow of patients with MDS [25]. In addition, CDC25C is part of the common deleted region in 5q- syndrome, the only subtype of MDS that has a defined cytogenetic cause and effective chemotherapy treatment [48–51]. Furthermore, haploinsufficiency of CDC25C is required for effective lenalidomide treatment of patients with 5q- syndrome [52]. RT-PCR analysis of CDC25C revealed a distinctive change in the levels of the C5 isoform, which lacks exons 3, 5, and 6, relative to C1 among the SRSF2 mutants. Specifically, expression of the MDS-associated point mutants (SRSF2^P95H^, SRSF2^P95L^, and SRSF2^P95R^) each caused an increase in the CDC25C C5/C1 ratio (Fig. 4b, c). In contrast, expression of SRSF2^WT^ or the deletion mutants did not affect this ratio.

We then considered that this alternative splicing change may cause expression of a truncated CDC25C protein. Western blotting using an anti-CDC25C antibody, specific for the C-terminus of the protein, detected the ~60 kDa CDC25C C1 protein and an increased expression of a smaller protein isoform that corresponds to the expected ~45 kDa CDC25C C5 protein (Fig. 4d, e). This result indicated that expression of SRSF2^P95H^, SRSF2^P95R^, or SRSF2^P95L^ causes a splicing change of CDC25C mRNA, whereas expression of the SRSF2 deletion mutants or the wild-type protein does not produce the alternative splicing change.

### CDC25C alternative splicing and the DNA damage response

**Fig. 5:**
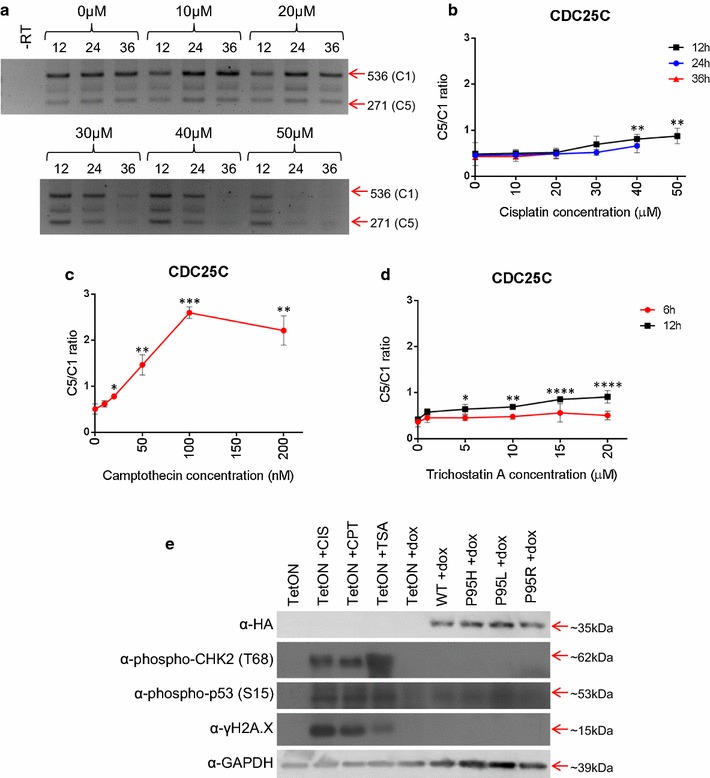
Drug-induced CDC25C alternative splicing. **a** Representative RT-PCR gel of alternative splicing of CDC25C in response to cisplatin treatment. (**b** to **d**) *Line graph* depicting average C5/C1 ratios for CDC25C in TF-1 TetON cells treated with **b** cisplatin, **c** CPT, and **d** TSA treated TF-1 TetON cells. Statistics are determined by two-way ANOVA with comparison to 0 µM (n = 4). **e** Western blot of HA-tagged protein, phosphorylated CHK2, phosphorylated p53, and GAPDH after no treatment (*lane 1*), 12 h 50 µM CIS (*lane 2*), 6 h 200 nM CPT (*lane 3*), 12 h 20 µM TSA (*lane 4*), or 48 h doxycycline treatment (*lanes 5*–*9*). *Asterisks* are *p* values as in Fig. 1

The elevated ratio of CDC25C C5/C1 detected in our TF-1 SRSF2-P95 mutants, has also been reported to be induced in breast cancer cells by treatment with sublethal doses of DNA-damaging agents, such as cisplatin (CIS) and doxorubicin [31]. To test whether DNA damaging agents would also alter alternative splicing of CDC25C in hematopoietic cells like TF-1, we treated TF-1 cells with various concentrations of CIS. As shown in Fig. 5a, b the ratio of C5/C1 indeed increased when cells were treated with 40–50 μM of CIS for 12 h. CIS forms DNA crosslinks that can cause double-stranded breaks that activate the DNA damage response via the ATM pathway [53–55]. We also tested other drugs that induce the ATM DNA damage response pathway: camptothecin (CPT), a topoisomerase I poison that causes chromosomal breaks [56–58], and trichostatin A (TSA), a histone deacetylase inhibitor [59, 60]. Similar to the effects observed with CIS treatment, CPT at 20–200 nM for 6 h or TSA at 5–20 μM for 12 h also caused an increase of the C5 splice variant of CDC25C (Fig. 5c, d; Additional file 6: Figure S5a, b). We noticed that some RT-PCR signals were weak or barely detectable—this was evident in RNA samples from cells treated with high drug doses or extended time—likely resulted from low RNA yields under those conditions. Similar weak RT-PCR signals were also observed and used to quantify the ratio of C5 to C1 in heavily treated cells by Albert et al. [31]. We also analyzed the protein levels of the CDC25C isoforms by immune-blotting and found evidence consistent with a percent increase of the C5 protein isoform in cells treated with CIS, CPT, or TSA at a dosage that caused CDC25C alternative splicing (Additional file 6: Figure S5c, d).

Since agents that activate the ATM-mediated DNA damage response cause a similar splicing change (elevated CDC25C C5/C1) as the MDS-associated SRSF2 mutants, we wondered whether these point mutants may also activated the ATM-mediated DNA damage response. To test this, we examined whether the phosphorylation of ATM substrates (p53, CHK2, and H2A.X [61–68]) increases in the drug-treated cells and/or in the SRSF2 mutant expressing cells. Cell lysates were analyzed using Western blotting for phospho-p53 and phospho-pCHK2. Treatment of the TF-1 TetON cell line with CIS, CPT, or TSA led to an increase in both phospho-p53 and phospho-CHK2 compared to untreated TF-TetON cells, as expected (Fig. 5e). However, ectopically expressing SRSF2 in TF-1 cells did not show a significant increase in phospho-p53 or phospho-CHK2 compared to doxycyline-treated TF-1 TetON, regardless of whether the SRSF2 was WT or a P95 mutant. We also tested levels of γH2A.X phosphorylation, a general marker for genome instability [69], in each of these cell lines. Again, CIS, CPT, and TSA treatment each increased γH2A.X levels while none of the SRSF2 cell lines had an increase in γH2A.X (Fig. 5e; Additional file 7: Figure S6). In addition, by propidium iodide staining, none of the MDS-related point mutants induced G2/M arrest (Additional file 8: Figure S7a). To test whether apoptosis played a role in CDC25C alternative splicing, we added caspase inhibitor zVAD to block apoptosis in cells expressing SRSF2-P95 mutants. The ratio between splice variant C5 and C1 increased when SRSF2-P95 mutant expression was induced as expected; however, the addition of zVAD had no observable effect on CDC25C alternative splicing regardless whether SRSF2-P95 mutant expression was induced or not (Additional file 8: Figure S7B, C). This result indicated that apoptosis, although occurred in SRSF2-P95 mutant cells, was not responsible for the CDC25C alternative splicing. Taken together, it appears that the induction of CDC25C alternative splicing by MDS-related SRSF2 point mutations does not require activation of classical ATM DNA damage response pathway.

## Discussion

Known functions of SRSF2 include regulation of constitutive and alternative splicing and maintenance of genome stability through the prevention of transcription-related R-loops [2, 3, 70]. Nearly half of all patients with MDS have mutation in a gene that regulates splicing. One of the most common mutations is the point mutation of proline 95 of SRSF2 to histidine, leucine, or arginine, which occurs in 15–20 % of all MDS patients. Recent research has shown that this mutation affects the RNA recognition specificity of SRSF2 and that the P95H mutation in a mouse model correlates with a phenotype similar to MDS [28–30]. However, the effect of SRSF2 mutation on distinct alternative splicing events remains poorly understood. In this study, we show that MDS-related SRSF2 mutants act similar to wildtype SRSF2 in many ways, including phosphorylation and splicing of a select group of alternative splicing events. However, there is an alternative splicing change in CDC25C that is unique to SRSF2^P95^ mutants. Chemotherapeutics that induce the classical ATM DNA damage response, including cisplatin, camptothecin, and trichostatin A, also induce an identical alternative splicing change in CDC25C.

Recent research has shown that various types of DNA damage can lead to post-translational modification of RNA-binding proteins, many of which have roles in the regulation of RNA splicing [71–75]. In fact, cisplatin treatment has been shown to cause increased translocation of SR protein kinases SRPK1 and SRPK2 to the nucleus leading to hyperphosphorylation of SRSF2 and reducing acetylation of SRSF2 [11]. In addition, activation of ATM- or ATR-mediated DNA damage response pathway has been shown to result in multiple changes in specific alternative splicing events, such as alternative splicing of apoptotic genes (BCL-X and caspase-8) [11, 76], DNA damage response genes (CHK1 and CHK2) [77, 78], and regulators of RNA splicing itself (TRA2, U2AF1, SRSF2 and SRSF1) [79–82]. However, the full changes in alternative splicing and their consequences for the DNA damage response still remain largely unknown.

Although it is known that CDC25C alternative splicing occurs after treatment with genotoxic drugs, the affects of this alternative splicing on the DNA damage response (DDR) and cell fate is still undetermined. CDC25C protein undergoes various post-translation modifications during the DDR in order to either induce the G2/M checkpoint or apoptosis. One of the major post-translational modifications is phosphorylation at Ser216 by CHK2, which leads to interaction of CDC25C with the 14-3-3 complex and subsequent translocation of the CDC25C protein to the cytoplasm [83]. Without CDC25C present in the nucleus to activate CDK1 by dephosphorylation at Tyr15, the cell enters G2/M arrest. The C5 isoform of CDC25C is upregulated during S phase, a cell cycle phase during which DNA repair frequently occurs due to DNA replication; however, the role of the C5 isoform in the S phase is not clear [84]. The alternative splicing of CDC25C, and its related protein CDC25A, is notable because it occurs primarily in the region of each transcript that encodes for the regulatory domain of the protein while leaving the catalytic domain intact (Fig. 4a) [84]. It is possible that alternative splicing of CDC25C, in conjunction with post-translational modification, plays an important regulatory role in cell fate after DNA damage. Interestingly, our results showed that the SRSF2 P95 point mutants cause the same alternative CDC25C splicing change without activating conventional DDR.

CDC25C is part of the commonly deleted region in 5q-syndrome, and effective treatment of 5q- syndrome with lenalidomide requires haploinsufficiency of CDC25C [48–52]. Furthermore, alternative splicing of CDC25C has already been observed in bone marrow samples from patients with MDS [25]. In addition, mutations in DNA damage response genes, such as p53 (*TP53*) and ATM, are found in MDS patients without 5-q deletion [85], and MDS-related U2AF1 mutations lead to alternative splicing in the DNA damage response genes ATR and FANCA [86]. Therefore, it appears that CDC25C may be recurrently misregulated in MDS through gene deletion, aberrant DNA damage response, or aberrant splicing factors like SRSF2-P95 mutants and U2AF1 mutants.

## Conclusion

In conclusion, our data support a model where MDS-related SRSF2 mutants lead to alternative splicing of CDC25C that increases the percentage of the C5 isoform among all the CDC25C variants. This alternative splicing event can also be induced by cisplatin, camptothecin, or trichostatin A treatment. However, the SRSF2 point mutants do not lead to phosphorylation of CHK2 or p53, which is readily detected in the drug-treated cells. Therefore, SRSF2P95H/L/R-mediated CDC25C alternative splicing changes do not require activation of the classical ATM DDR. The mechanism of CDC25C alternative splicing and its downstream effects may be pertinent to the role of SRSF2′s point mutation in the development of myelodysplastic syndrome.

## Additional files

10.1186/s12867-016-0071-y Supplementary information: supplemental figure legends, supplemental references, and Table S1.
10.1186/s12867-016-0071-y HA-tagged SRSF2 protein expression in uninduced TF-1 cell lines.
10.1186/s12867-016-0071-y Apoptosis, subcellular localization, and cell proliferation of SRSF2 mutants.
10.1186/s12867-016-0071-y Alternative splicing of apoptosis genes.
10.1186/s12867-016-0071-y Alternative splicing of genes from a previous study using SRSF2 depletion.
10.1186/s12867-016-0071-y Alternative splicing of CDC25C in TF-1 cells treated with CIS, CPT, or TSA.
10.1186/s12867-016-0071-y DNA damage in SRSF2 mutant cell lines.
10.1186/s12867-016-0071-y Cell cycle analysis of SRSF2 mutant cells and the effect of an apoptosis inhibitor on CDC25C alternative splicing.

## Abbreviations

SRSF2: serine–arginine rich splicing factor 2
MDS: myelodysplastic syndrome
CDC25C: cell division cycle 25C
ATM: ataxia telangiectasia mutated
RRM: RNA recognition motif
RS: serine–arginine rich domain
HNG: hinge region
NRS: nuclear retention signal
CMML: chronic myelomonocytic leukemia
