# Supplementary material for: Myelodysplasia-associated mutations in serine/arginine-rich splicing factor SRSF2 lead to alternative splicing of CDC25C
Source: BMC Mol Biol. 2016 Aug 23;17(1):18. doi: 10.1186/s12867-016-0071-y (PMC4994158; doi:10.1186/s12867-016-0071-y)
Supplement: Supplementary file 1 — 10.1186/s12867-016-0071-y Supplementary information: supplemental figure legends, supplemental references, and Table S1. [file 12867_2016_71_MOESM1_ESM.docx]

**Supplementary Information**

**Myelodysplasia-associated mutations in serine/arginine-rich splicing factor SRSF2 lead to alternative splicing of CDC25C**

Lindsey Skrdlant^1^, Jeremy M. Stark^2^, and Ren-Jang Lin^3,^*

^1^Irell & Manella Graduate School of Biological Sciences and Department of Molecular and Cellular Biology, Beckman Research Institute of the City of Hope, Duarte, CA 91010, USA; [LSkrdlant@coh.org](mailto:LSkrdlant@coh.org)

^2^Irell & Manella Graduate School of Biological Sciences and Department of Cancer Genetics and Epigenetics, Beckman Research Institute of the City of Hope, Duarte, CA 91010, USA; [JStark@coh.org](mailto:JStark@coh.org)

^3^Irell & Manella Graduate School of Biological Sciences and Department of Molecular and Cellular Biology, Beckman Research Institute of the City of Hope, Duarte, CA 91010, USA; [RLin@coh.org](mailto:RLin@coh.org)

*Corresponding author: 1-626-301-8286; [RLin@coh.org](mailto:RLin@coh.org)

Running Title: SRSF2-P95 mutations and CDC25C alternative splicing

**Supplemental Figure Legends**

**Supplemental Figure 1.** A) Western blot of protein isolated from cell lines without doxycycline induction. B) Average HA-tagged protein expression in non-induced cell lines, normalized to GAPDH expression (n=5). The relative expression is a comparison to the SRSF2-WT with dox induction.

**Supplemental Figure 2. Apoptosis, subcellular localization, and cell proliferation of SRSF2 mutants.** A) Quantification of HA tagged protein from confocal images shown in Fig. 2A (n=25). Statistical analysis consisted of one-way ANOVA with comparison to WT. Asterisks are *p*-values as in Fig. 1. B) Representative FACS plots for apoptosis assays quantified in Fig. 2B showing gating for early/middle apoptosis and late apoptosis. The x-axis represents FITC staining, and the y-axis represents PI staining. C) Cell proliferation analysis of the TF-1 TetON parental cell line, SRSF2^WT^, and MDS-related SRSF2 P95 mutants using CFSE staining (n=4). No statistical significance was observed using one-way ANOVA with comparison to the TF-1 TetON parental cell line.

**Supplemental Figure 3. Alternative splicing of apoptosis genes.** A) Representative gel from RT-PCR of BCL-X after 48h treatment with 2 μg/ml doxycycline. B) Bar graphs depicting % of the additional exon sequence spliced in. C) Representative gel from RT-PCR of Caspase-8 after 48h treatment with 2 μg/ml doxycycline. D) Bar graphs depicting % of the additional exon sequence spliced in. E) Representative gel from RT-PCR of Caspase-9 after 48h treatment with 2 μg/ml doxycycline. F) Bar graphs depicting % of the cassette exon spliced in. No statistical significance of any of these alternative splicing events, based on two-way ANOVA with comparison to SRSF2^WT^ (n=4).

**Supplemental Figure 4. Alternative splicing of genes from a previous study using SRSF2 depletion.** A) Representative gel from RT-PCR of BAP1 after 48h treatment with 2 μg/ml doxycycline. B) Bar graphs depicting % of the cassette exon spliced in. No statistical significance based on 2way ANOVA with comparison to SRSF2^WT^ (n=4). C) Representative gel from RT-PCR of TRA2A after 48h treatment with 2 µg/ml doxycyline. D) Bar graphs depicting % of the cassette exon spliced in. No statistical significance based on 2way ANOVA with comparison to SRSF2^WT^ (n=4).

**Supplemental Figure 5.** A) Representative gel from RT-PCR of CDC25C after 6h treatment with CPT. B) Representative gel from RT-PCR of CDC25C after 6h or 12h treatment with TSA. C) Western blot depicting CDC25C protein expression in the TF-1 TetON parental cell line with no treatment or in the presence of CIS (50µM for 12h), CPT (200nM for 6h), or TSA (20µM for 12h) treatments. D) Graph depicting the Western results shown in C for CDC25C protein expression. Statistics are based on two-way ANOVA with comparison to untreated TF-1 TetON cells (TetON) (n=5). Asterisks are *p*-values as in Fig. 1. A significant increase of total CDC25C protein in the TSA-treated sample may be resulted from a TSA-induced G2/M arrest, which has been reported to increase the amount of total CDC25C protein [[1](#_ENREF_1), [2](#_ENREF_2)].

**Supplemental Figure 6. DNA damage in SRSF2 mutant cell lines.** Representative immunofluorescence images of SRSF2HA cell lines after 48h 2µg/ml doxycycline treatment. CellMask Orange stained cytoplasm, DAPI stained DNA in the nucleus, anti-γH2AX stained DNA damage foci, and anti-HA stained HA-tagged SRSF2 protein. TF-1 TetON cell line treated for 12h with 50µM CIS was used as a positive control for DNA damage.

**Supplemental Figure 7.** A) Cell cycle analysis of TF-1 TetON cell lines without treatment or with treatment by 50µM CIS (12h), 200nM CPT (6h), or 20µM TSA (12h), and SRSF2 wildtype and MDS-related SRSF2 point mutants with 48h 2µg/ml doxycycline induction. Statistics are based on two-way ANOVA with comparison to untreated TF-1 TetON cells (TetON) (n=4). Asterisks are *p*-values as in Fig. 1. B) Representative RT-PCR gels of CDC25C alternative splicing in cell lines grown for 48h in the absence of any treatment, 20μM zVAD treatment alone, 2µg/ml doxycycline treatment alone, or combined doxycycline and zVAD treatment. C) Bar graph representing C5/C1 ratio of CDC25C alternative splicing for the treatment groups outlined in B. Statistics are based on two-way ANOVA with comparison to the untreated (-dox) sample within each cell line. Asterisks are *p*-values as in Fig. 1 (n=4). Z-VAD has been reported to prevent apoptosis in hematopoietic cell lines similar to the TF-1 cells and in megakaryocyte in dosages comparable to what is used here [[3-10](#_ENREF_3)].

**Supplemental References**

1. Zhan W, Han T, Zhang C, Xie C, Gan M, Deng K, Fu M, Wang JB: **TRIM59 Promotes the Proliferation and Migration of Non-Small Cell Lung Cancer Cells by Upregulating Cell Cycle Related Proteins**. *PloS one* 2015, **10**(11):e0142596.

2. Wang YR, Xu Y, Jiang ZZ, Guerram M, Wang B, Zhu X, Zhang LY: **Deoxypodophyllotoxin induces G2/M cell cycle arrest and apoptosis in SGC-7901 cells and inhibits tumor growth in vivo**. *Molecules* 2015, **20**(1):1661-1675.

3. Mundle SD, Reza S, Ali A, Mativi Y, Shetty V, Venugopal P, Gregory SA, Raza A: **Correlation of tumor necrosis factor alpha (TNF alpha) with high Caspase 3-like activity in myelodysplastic syndromes**. *Cancer letters* 1999, **140**(1-2):201-207.

4. Yang H, Miller WM, Papoutsakis ET: **Higher pH promotes megakaryocytic maturation and apoptosis**. *Stem cells* 2002, **20**(4):320-328.

5. Buss JL, Neuzil J, Gellert N, Weber C, Ponka P: **Pyridoxal isonicotinoyl hydrazone analogs induce apoptosis in hematopoietic cells due to their iron-chelating properties**. *Biochemical pharmacology* 2003, **65**(2):161-172.

6. Holme JA, Morrison E, Samuelsen JT, Wiger R, Lag M, Schwarze PE, Bernhoft A, Refsnes M: **Mechanisms involved in the induction of apoptosis by T-2 and HT-2 toxins in HL-60 human promyelocytic leukemia cells**. *Cell biology and toxicology* 2003, **19**(1):53-68.

7. Albee L, Perlman H: **E. coli infection induces caspase dependent degradation of NF-kappaB and reduces the inflammatory response in macrophages**. *Inflammation research : official journal of the European Histamine Research Society [et al]* 2006, **55**(1):2-9.

8. Prochazkova J, Stixova L, Soucek K, Hofmanova J, Kozubik A: **Monocytic differentiation of leukemic HL-60 cells induced by co-treatment with TNF-alpha and MK886 requires activation of pro-apoptotic machinery**. *European journal of haematology* 2009, **83**(1):35-47.

9. Eguchi R, Kubo S, Ohta T, Kunimasa K, Okada M, Tamaki H, Kaji K, Wakabayashi I, Fujimori Y, Ogawa H: **FK506 induces endothelial dysfunction through attenuation of Akt and ERK1/2 independently of calcineurin inhibition and the caspase pathway**. *Cellular signalling* 2013, **25**(9):1731-1738.

10. Avanzi MP, Izak M, Oluwadara OE, Mitchell WB: **Actin inhibition increases megakaryocyte proplatelet formation through an apoptosis-dependent mechanism**. *PloS one* 2015, **10**(4):e0125057.

**Additional file 1: Table S1.**

| Primer set | Sequence | Annealing Temp and Time | Extension Time | # Cycles |
| --- | --- | --- | --- | --- |
| SRSF2 (genotyping) | F = 5’-GTGGACAACCTGACCTACCG-3’  R = 5’-CCTCAGCCCCGTTTACCT-3’ | 54°C; 30s | 30s | 35 |
| SRSF2 3’UTR | F = 5’-GAAGGAGCGGTGTCCTCTTA-3’  R = 5’-AGCACTCTGCACTGCAACAC-3’ | 54°C; 30s | 60s | 27 |
| Caspase 8 | F = 5’-GGGATACTGTCTGATCATCAAC-3’  R = 5’-GGAGAGGATACAGCAGATGAA-3’ | 56°C; 30s | 60s | 27 |
| Caspase 9 | F = 5’-AGACCAGTGGACATTGGTTC-3’  R = 5’-GGTCCCTCCAGGAAACAAA-3’ | 59°C; 60s | 120s | 27 |
| BCL-X | F = 5’-ATGTCTCAGAGCAACCGGGA-3’  R = 5’-TCACTTCCGACTGAAGAGTG-3’ | 55°C; 60s | 60s | 27 |
| BAP1 | F = 5’-ACGTCCGTGATTGATGATGA-3’  R = 5’-GTAGACCTTCAGCCCATCCA-3’ | 53°C; 30s | 60s | 27 |
| TRA2A | F = 5’-AAAACAACTTCGAGGGCAGA-3’  R = 5’-TCCATTTGCCCTTTCCATAG-3’ | 50°C; 30s | 60s | 27 |
| CDC25C | F = 5’-CTCCTGGAGAGAGACACTTCCTTTAC-3’  R = 5’-CCACTTCTGCTCACCTTTGCTTCTTG-3’ | 61°C; 60s | 90s | 27 |
