# Supplementary figures and images for: Myelodysplasia-associated mutations in serine/arginine-rich splicing factor SRSF2 lead to alternative splicing of CDC25C
Source: BMC Mol Biol. 2016 Aug 23;17(1):18. doi: 10.1186/s12867-016-0071-y (PMC4994158; doi:10.1186/s12867-016-0071-y)

**A****-dox**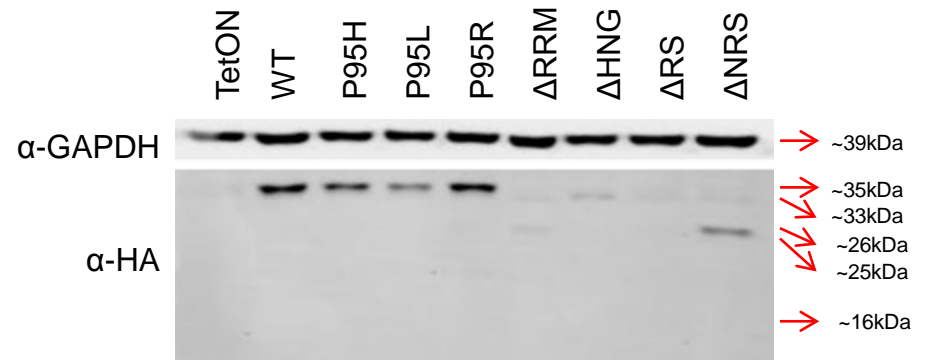**B****HA-tagged SRSF2 -dox**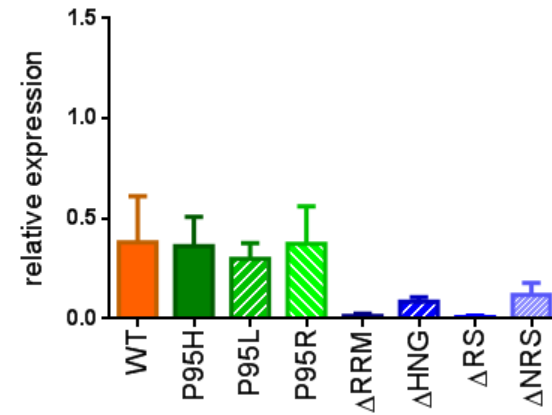

Supplement: Supplementary file 2 — 10.1186/s12867-016-0071-y HA-tagged SRSF2 protein expression in uninduced TF-1 cell lines. [file 12867_2016_71_MOESM2_ESM.pdf]

**A** Subcellular localization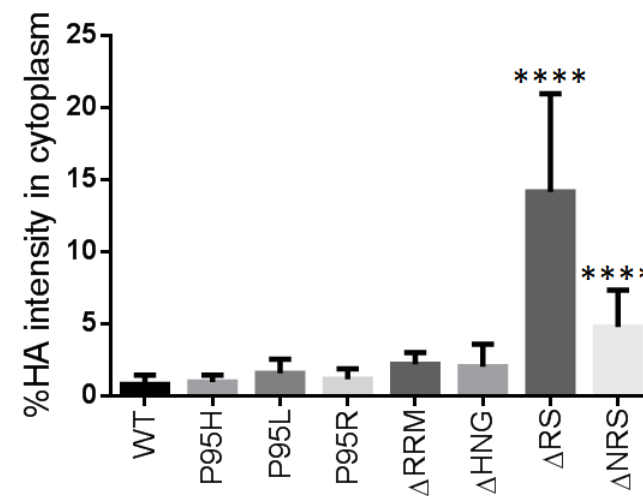**B**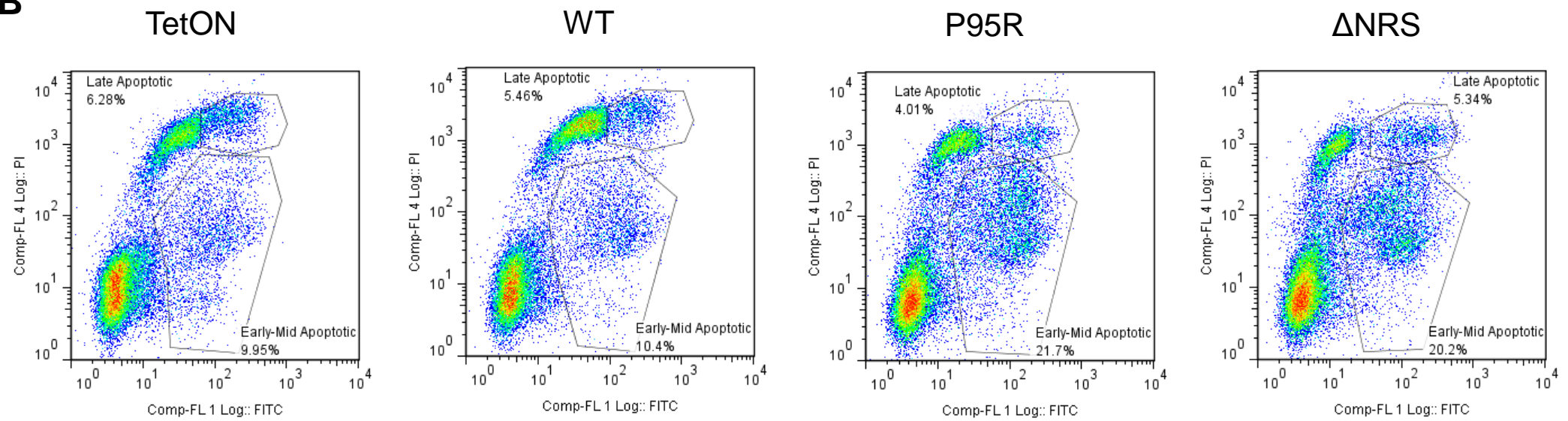**C**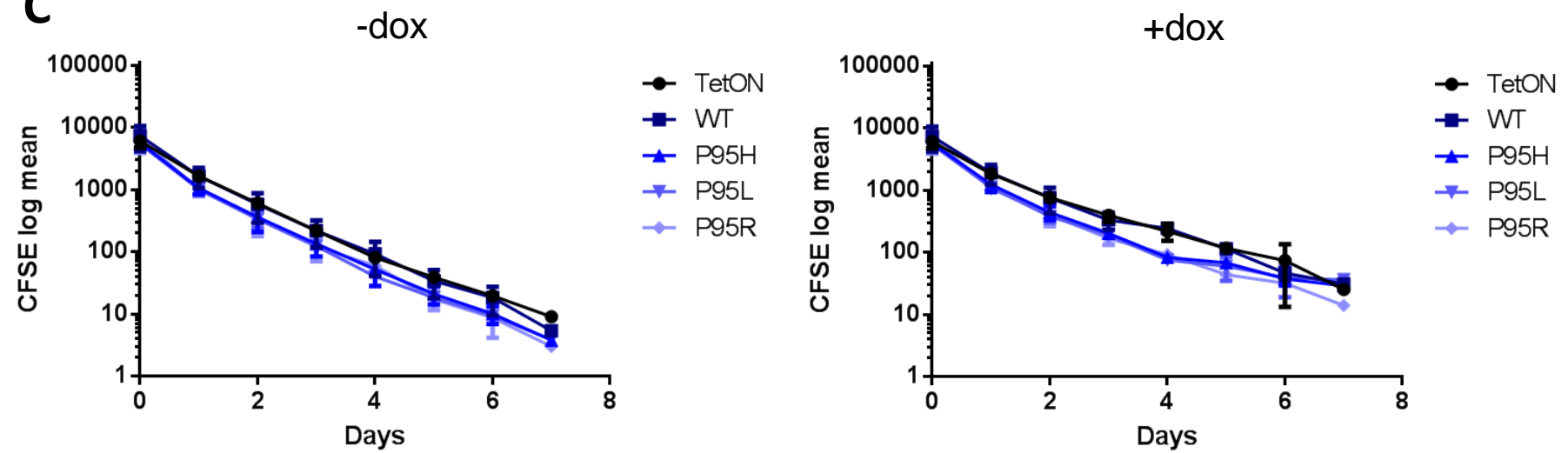

Supplement: Supplementary file 3 — 10.1186/s12867-016-0071-y Apoptosis, subcellular localization, and cell proliferation of SRSF2 mutants. [file 12867_2016_71_MOESM3_ESM.pdf]

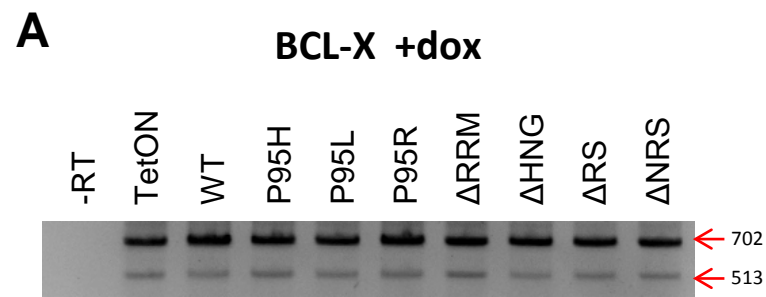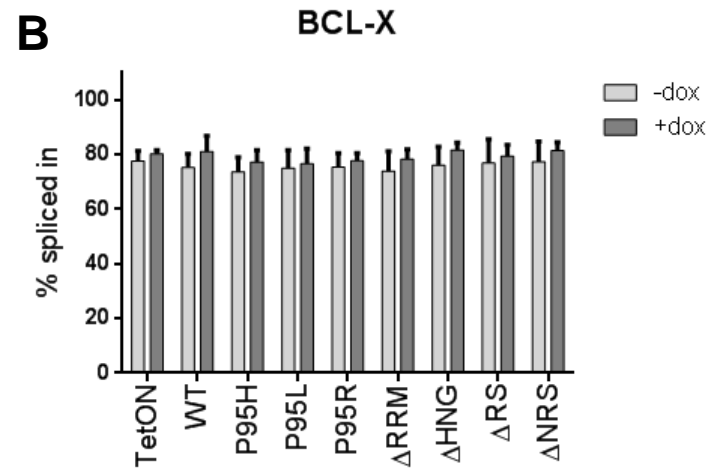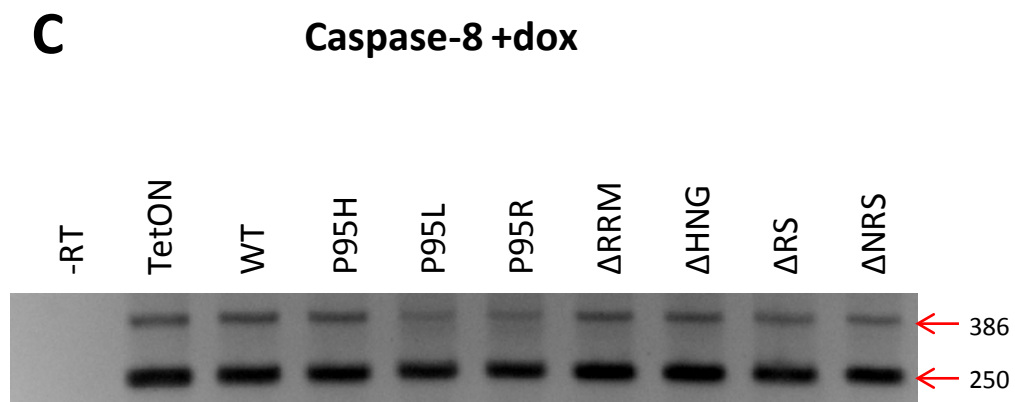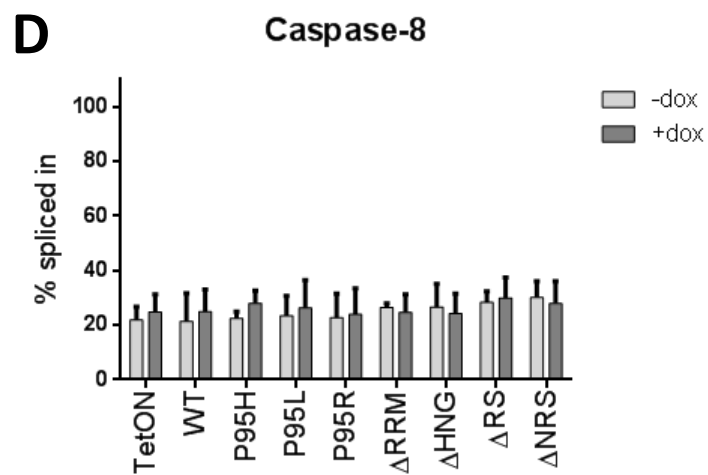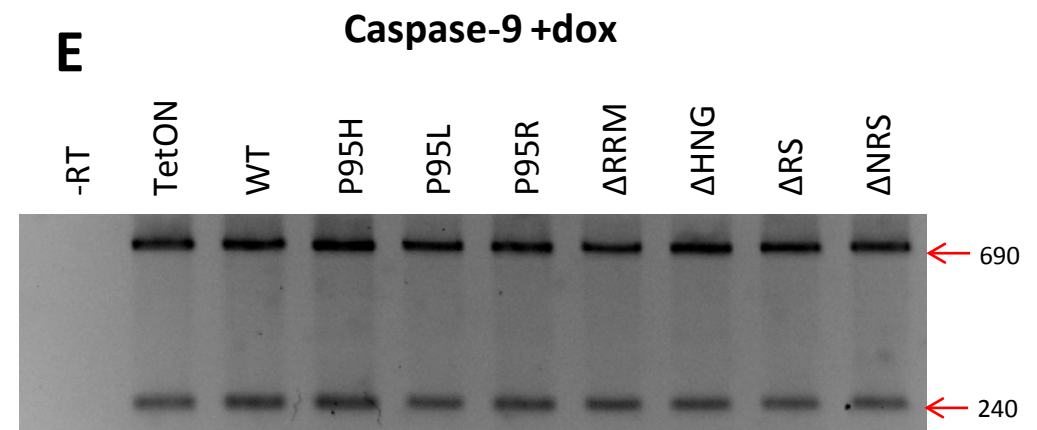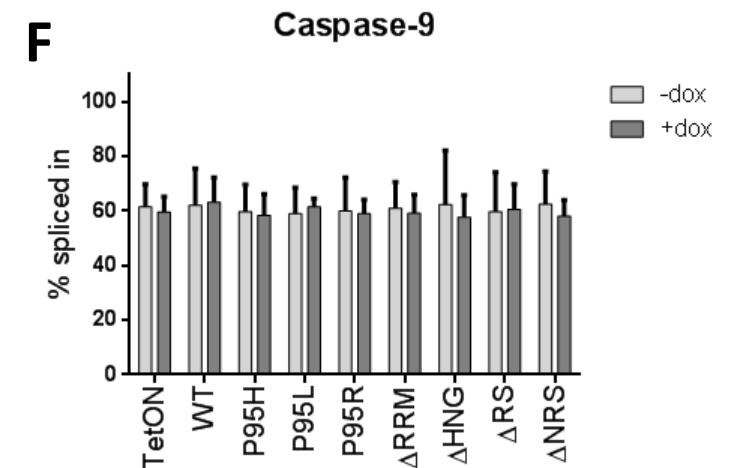

Supplement: Supplementary file 4 — 10.1186/s12867-016-0071-y Alternative splicing of apoptosis genes. [file 12867_2016_71_MOESM4_ESM.pdf]

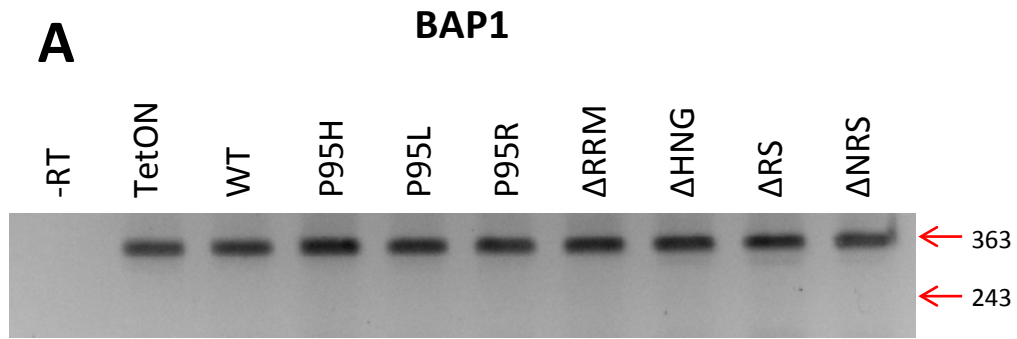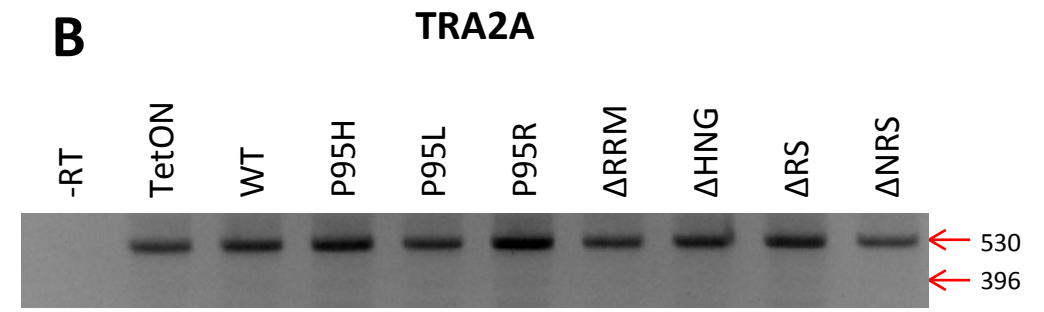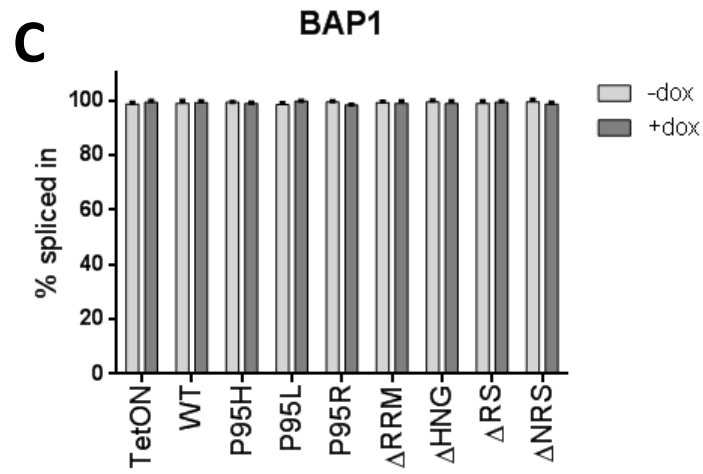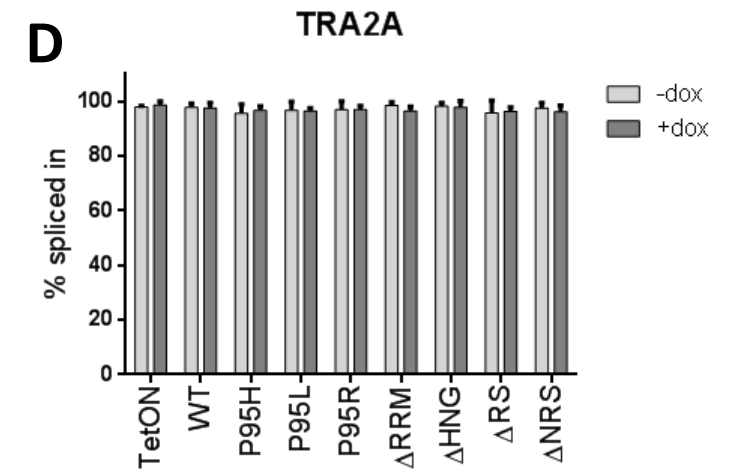

Supplement: Supplementary file 5 — 10.1186/s12867-016-0071-y Alternative splicing of genes from a previous study using SRSF2 depletion. [file 12867_2016_71_MOESM5_ESM.pdf]

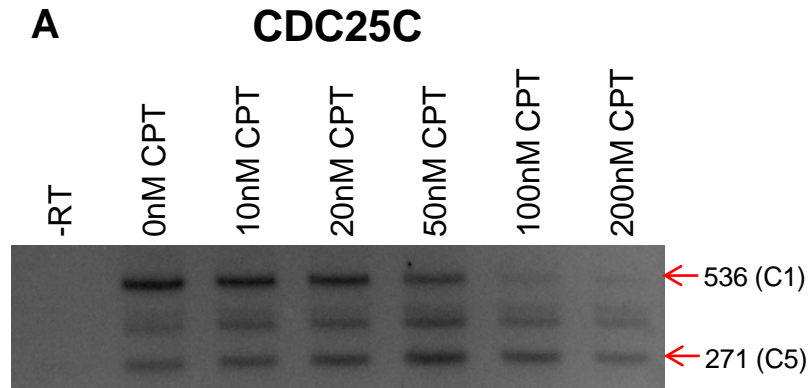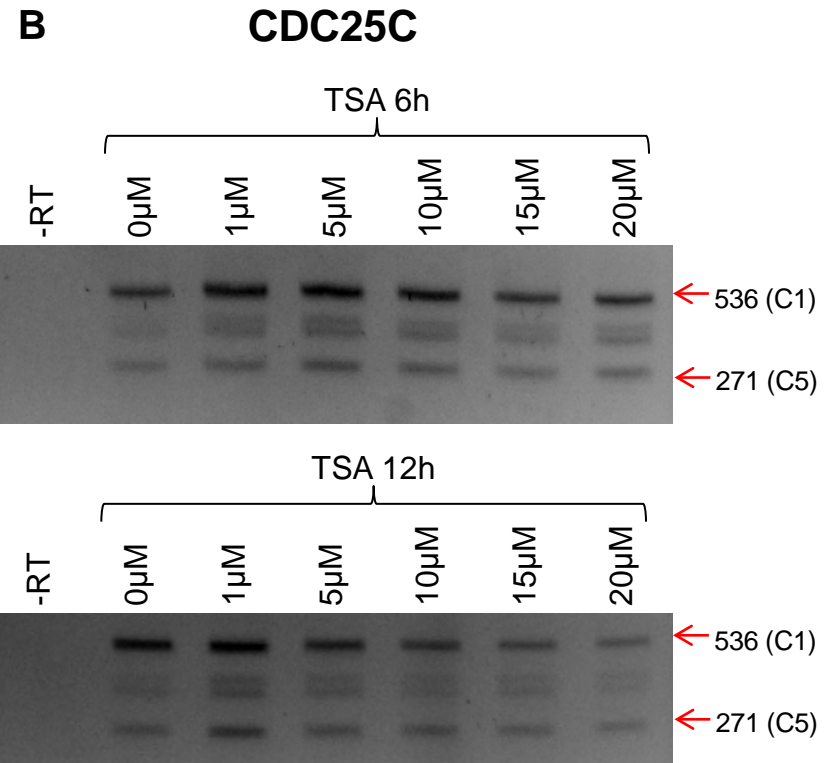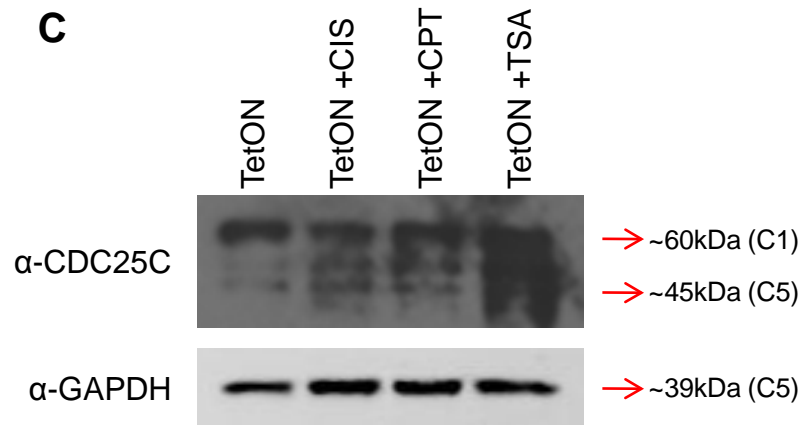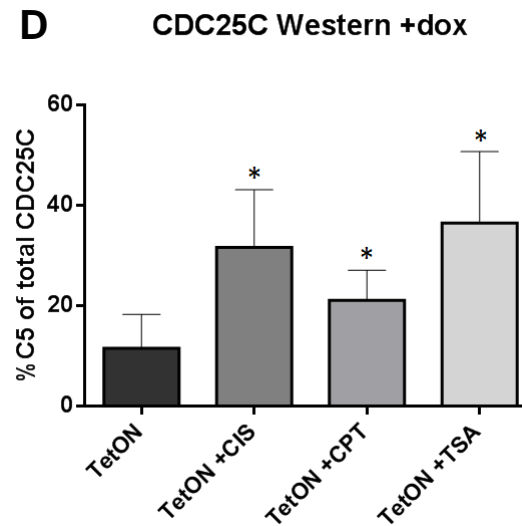

Supplement: Supplementary file 6 — 10.1186/s12867-016-0071-y Alternative splicing of CDC25C in TF-1 cells treated with CIS, CPT, or TSA. [file 12867_2016_71_MOESM6_ESM.pdf]

Supplemental Figure 6, Skrdlant et al. 2016

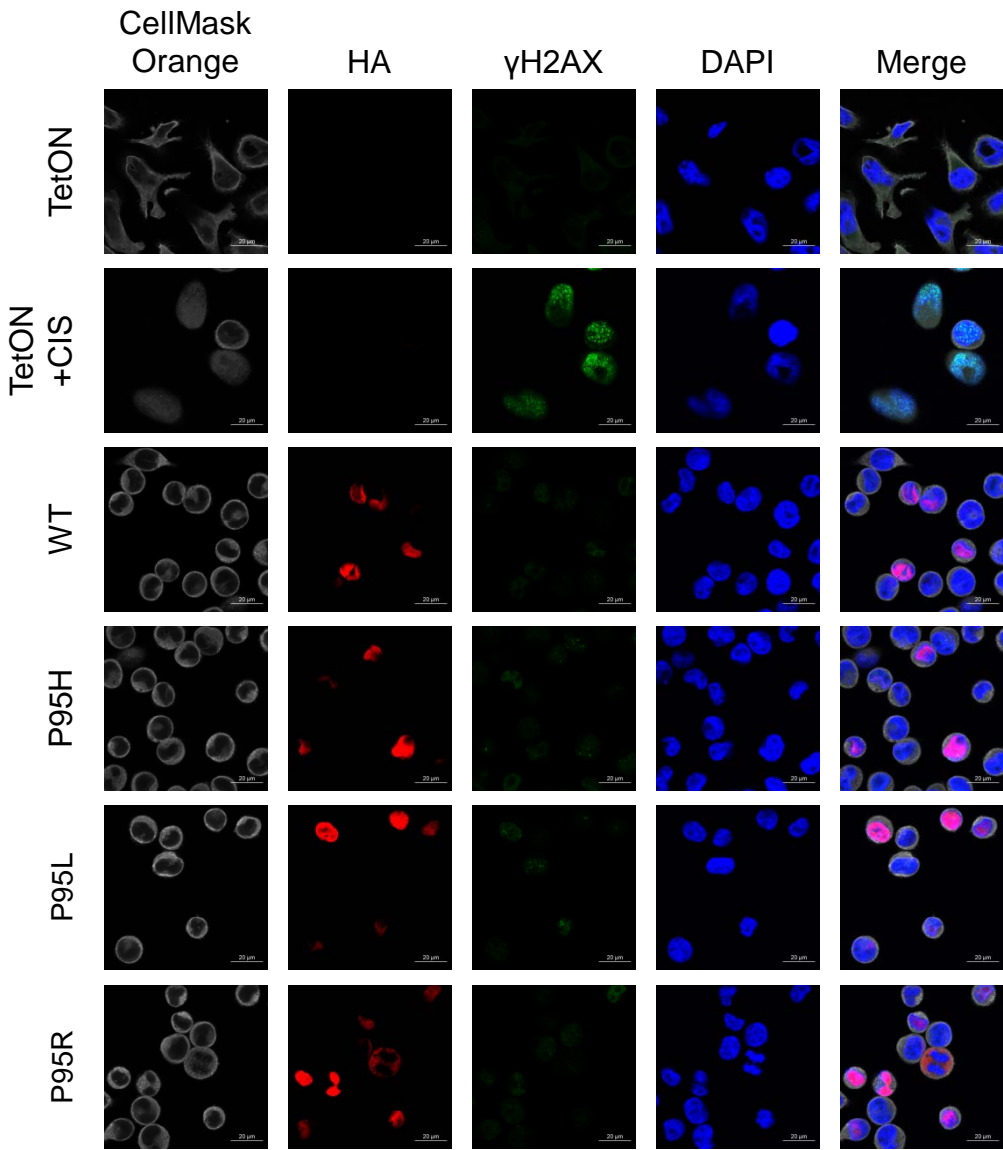

Supplement: Supplementary file 7 — 10.1186/s12867-016-0071-y DNA damage in SRSF2 mutant cell lines. [file 12867_2016_71_MOESM7_ESM.pdf]

**A****Cell Cycle Analysis**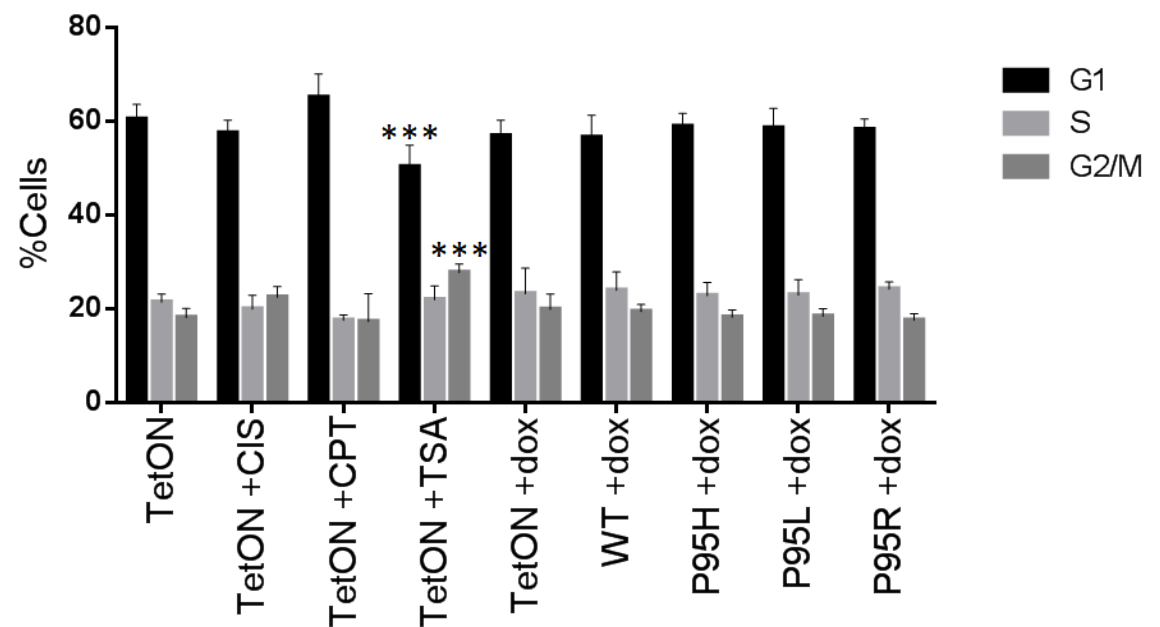**B****CDC25C -dox**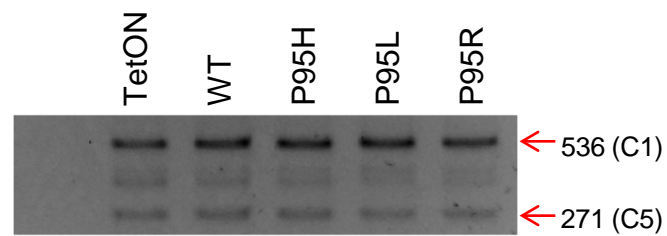**CDC25C -dox +zVAD**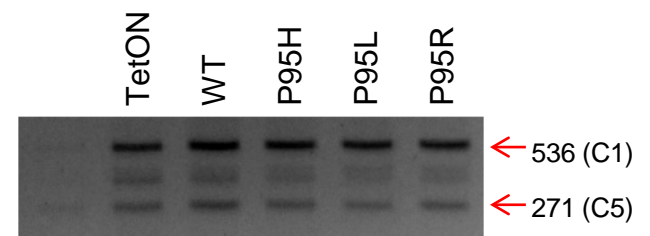**CDC25C +dox**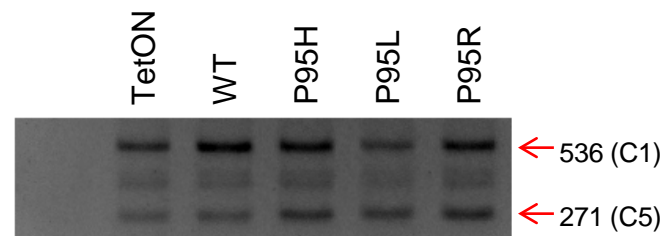**CDC25C +dox +zVAD**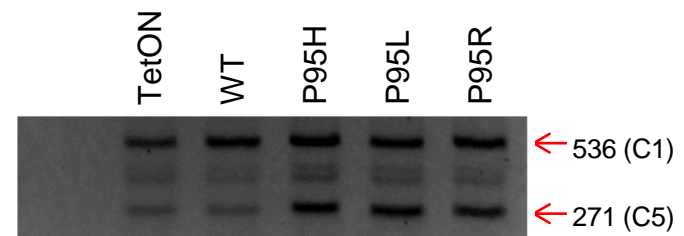**C****CDC25C in TF-1 TetON cells**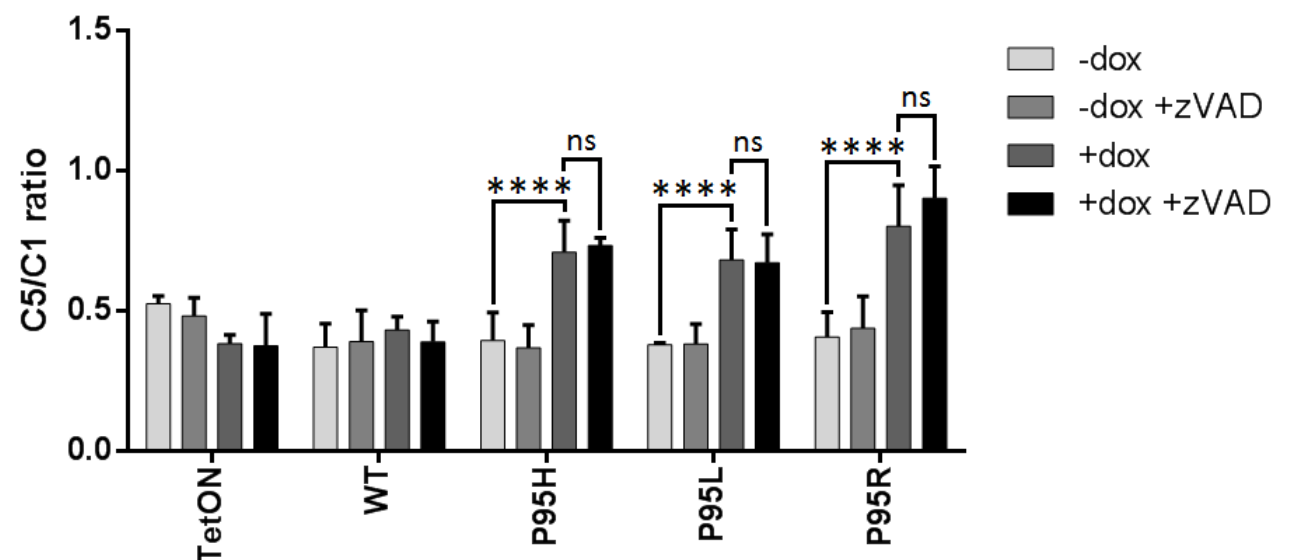

Supplement: Supplementary file 8 — 10.1186/s12867-016-0071-y Cell cycle analysis of SRSF2 mutant cells and the effect of an apoptosis inhibitor on CDC25C alternative splicing. [file 12867_2016_71_MOESM8_ESM.pdf]
